# Characterization of the rainbow trout spleen transcriptome and identification of immune-related genes

**DOI:** 10.3389/fgene.2014.00348

**Published:** 2014-10-14

**Authors:** Ali Ali, Caird E. Rexroad, Gary H. Thorgaard, Jianbo Yao, Mohamed Salem

**Affiliations:** ^1^Department of Biology, Middle Tennessee State UniversityMurfreesboro, TN, USA; ^2^Department of Zoology, Faculty of Science, Benha UniversityBenha, Egypt; ^3^The National Center for Cool and Cold Water Aquaculture, United States Department of Agriculture Agricultural Research ServiceLeetown, WV USA; ^4^School of Biological Sciences, Washington State UniversityPullman, WA, USA; ^5^Division of Animal and Nutritional Science, West Virginia UniversityMorgantown, WV, USA

**Keywords:** spleen transcriptome, annotation, KEGG, immune-related genes, spleen-specific genes, full-length cDNA

## Abstract

Resistance against diseases affects profitability of rainbow trout. Limited information is available about functions and mechanisms of teleost immune pathways. Immunogenomics provides powerful tools to determine disease resistance genes/gene pathways and develop genetic markers for genomic selection. RNA-Seq sequencing of the rainbow trout spleen yielded 93,532,200 reads (100 bp). High quality reads were assembled into 43,047 contigs. 26,333 (61.17%) of the contigs had hits to the NR protein database and 7024 (16.32%) had hits to the KEGG database. Gene ontology showed significant percentages of transcripts assigned to binding (51%), signaling (7%), response to stimuli (9%) and receptor activity (4%) suggesting existence of many immune-related genes. KEGG annotation revealed 2825 sequences belonging to “organismal systems” with the highest number of sequences, 842 (29.81%), assigned to immune system. A number of sequences were identified for the first time in rainbow trout belonging to Toll-like receptor signaling (35), B cell receptor signaling pathway (44), T cell receptor signaling pathway (56), chemokine signaling pathway (73), Fc gamma R-mediated phagocytosis (52), leukocyte transendothelial migration (60) and NK cell mediated cytotoxicity (42). In addition, 51 transcripts were identified as spleen-specific genes. The list includes 277 full-length cDNAs. The presence of a large number of immune-related genes and pathways similar to other vertebrates suggests that innate and adaptive immunity in fish are conserved. This study provides deep-sequence data of rainbow trout spleen transcriptome and identifies many new immune-related genes and full-length cDNAs. This data will help identify allelic variations suitable for genomic selection and genetic manipulation in aquaculture.

## Introduction

Teleost fish are the first class of vertebrates that have the elements of both innate and adaptive immune responses (Whyte, 2007). Innate immunity is more important in teleosts as the first line of defense due to the restrictions on adaptive immunity in suboptimal environments (Ullal et al., 2008). Teleost fish have no bone marrow or lymph nodes. Immunogenomics can be used in clarifying the origin and evolution of the immune systems (Kaiser et al., 2008).

The ability of fish to combat viral, bacterial or parasitic pathogen is affected by genetic factors. Thus, genetic selection can improve disease resistance and provide prolonged protection against pathogens (Skamene and Pietrangeli, 1991; Wiegertjes et al., 1996; Van Muiswinkel et al., 1999; Leeds et al., 2010; Wiens et al., 2013a). In addition, investigations on immune reactions in fish could aid in development of vaccines (Raida and Buchmann, 2009).

Rainbow trout (*Oncorhynchus mykiss*) are widely distributed and cultured aquaculture species used as a food and sport fish (Thorgaard et al., 2002). Additionally, this organism is used as a model species in many different fields of research such as cancer biology (Tilton et al., 2005), toxicology (Köllner et al., 2002), nutrition (Wong et al., 2013), evolutionary biology (Taylor et al., 2011), and immunology (Nya and Austin, 2011). Rainbow trout are larger than other fish model species, making them a source of large quantities of specific tissues and cells for immunological, biochemical and molecular analyses. Several genomic resources have been developed for research to help improve rainbow trout commercial production traits including disease resistance. The list includes clonal lines (Young et al., 1996; Thorgaard et al., 2002), BAC libraries (Palti et al., 2004), genetic linkage maps (Young et al., 1998; Sakamoto et al., 2000; Rexroad et al., 2008; Palti et al., 2011; Guyomard et al., 2012), microarrays (Salem et al., 2008), expressed sequence tags (ESTs) (Rexroad et al., 2003; Sánchez et al., 2009; Boussaha et al., 2012), single nucleotide polymorphisms (SNPs) (Sánchez et al., 2009; Amish et al., 2012; Boussaha et al., 2012; Houston et al., 2012; Salem et al., 2012; Christensen et al., 2013; Colussi et al., 2014; Palti et al., 2014), next-generation sequence read archives (SRA) and genome as well as transcriptome reference assemblies (Salem et al., 2010; Sanchez et al., 2011; Berthelot et al., 2014; Fox et al., 2014). Several studies have identified many immune-related key genes and gene networks (Thorgaard et al., 2002; Cerdà et al., 2008; Chiu et al., 2010; Zhang et al., 2011). Immunogenomic studies of rainbow trout have established an agricultural importance due to their direct and immediate contributions to the aquaculture industry (Thorgaard et al., 2002).

The spleen is a primary hematopoietic and peripheral lymphoid organ (Zapata et al., 2006; Mahabady et al., 2012). This organ has melano-macrophage centers for breakdown of aged erythrocytes, and T-like as well as B-like cells for antigen trapping. In addition, spleen has a role in antigen presentation and initiation of the adaptive immune response (Espenes et al., 1995; Zapata et al., 1997; Chaves-Pozo et al., 2005; Whyte, 2007; Alvarez-Pellitero, 2008). Positive genetic correlation exists between bacterial cold water disease resistance and spleen index in domesticated rainbow trout (Wiens et al., 2013b).

Transcriptomic profiling is useful in revealing spleen genes that are engaged in the innate and adaptive immune responses and expressed as a result of the presence of toxicants or infection (Pereiro et al., 2012). RNA-Seq (whole transcriptome sequencing) is an effective tool for identifying the functional complexity of transcriptomes, alternative splicing, non-coding RNAs, new transcription units and assembly of full-length genes (Xiang et al., 2010; Huang et al., 2011; Salem et al., 2012; Djari et al., 2013). This deep sequencing technology gives low background noise, aids in identifying allele-specific expression and reveals weakly expressed transcripts. Bioinformatics algorithms were developed facilitating transcriptomic profiling (Yang et al., 2012). Therefore, RNA-Seq is a valuable tool in studying functional complexity of the spleen transcriptome and identifying immune-relevant genes and signaling networks (Nie et al., 2012).

In this study, we aimed to (1) characterize the transcriptome of rainbow trout spleen and (2) identify spleen-specific and immune-relevant genes (including full-length cDNAs) that could be used to develop genetic markers for disease resistance. Identifying the networks associated with such genes will be helpful in generating new technologies to improve aquaculture (Takano et al., 2011).

## Results and discussion

The spleen transcriptome was sequenced from an apparently healthy single homozygous doubled-haploid fish from the Swanson clonal line, the same line used for BAC library construction (Palti et al., 2004) and sequencing both of the whole transcriptome (Salem et al., 2010) and the whole genome reference (Berthelot et al., 2014). A single doubled-haploid fish was used to help overcome the assembly complications associated with the tetraploid genome of the rainbow trout (Allendorf and Thorgaard, 1984). Spleen transcriptome RNA-Seq data were *de novo* assembled into contigs. Assembled contigs were analyzed and annotated to identify genes that are predominantly expressed in the spleen and genes that are involved in immune signaling pathways. Spleen sequencing data yielded a total of 93,532,200 reads with a read length of at least 100 bp. After filtration to remove the adaptors, low complexity reads and duplicates, 58,013,135 (62%) high quality reads (*Q* values > 33%) were obtained and assembled into 43,047 contigs with an average contig length of 1154 nt and N50 equal to 1306 nt.

### Functional annotation

Contigs were searched against the NCBI's non-redundant protein (NR) database using the BLASTX program with *E*-value of 1.0E-3. There were 26,333 (61.17%) contigs with hits to the NR database (Table S1). The contigs which had no hits [16,714 (38.83%)] may be attributed to non-coding RNAs, contig misassembles (Grabherr et al., 2011), limited information about protein sequences of related fish in the NCBI database or diverged sequences of rainbow trout due to partial genome duplication (Ravi and Venkatesh, 2008; Lee et al., 2011). Further work toward characterization of the non-coding RNAs is still needed.

**Figure 1:**
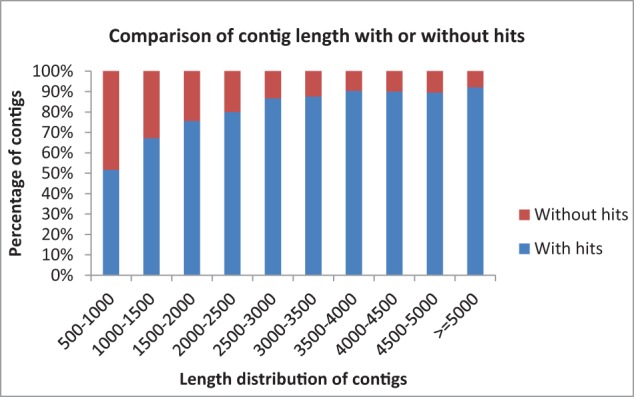
**Comparison of contig length with and without BLASTX hits to the NR database**.

Data statistics of the sequencing, assembly and annotations are presented in Table 1. A total of 13,780 (88.40%) of the contigs of more than 1000 bp in length had BLAST matches, whereas only 12,543 (51.72%) of contigs shorter than 1000 bp had BLAST hits (Figure 1). Short sequences may give false-negative results because they are not long enough to show sequence matches or may lack a representative protein domain (Wang et al., 2012). The identity distribution revealed that 70% of the contigs have greater than 80% similarity and 24% possess identity similarities between 60 and 80%. The *E*-value distribution of the top hits to the NR database showed that 28% of the assembled contigs showed significant homology to previously deposited sequences (less than 1.0E-50), and 72% ranged from 1.0E-50 to 0. The assembled contigs have been submitted to the NAGRP Aquaculture Genome Projects (http://www.animalgenome.org/repository/pub/MTSU2014.0811/).

**Table 1:** **Statistical summary of rainbow trout spleen sequencing, assembly and annotation**.

**Data generation and filtration**	
Total number of reads	93,532,200
Total number of duplicates	35,519,065 (38%)
Number of high quality reads	58,013,135 (62%)
**Assembly statistics**	
Number of bases	49,669,063
Number of long contigs (≥500)	43,047
Largest contig length (nt)	14,276
Smallest contig length (nt)	500
Average contig length (nt)	1154
N50 (nt)	1306
**Annotation**	
Total number of annotated contigs	43,047
Number of contigs with NR hits	26,333 (61.17%)
Number of contigs without NR hits	16,714 (38.83%)
Number of contigs with KEGG hits	7024 (16.32%)
Number of contigs without KEGG hits	36,023 (83.68%)

**Figure 2:**
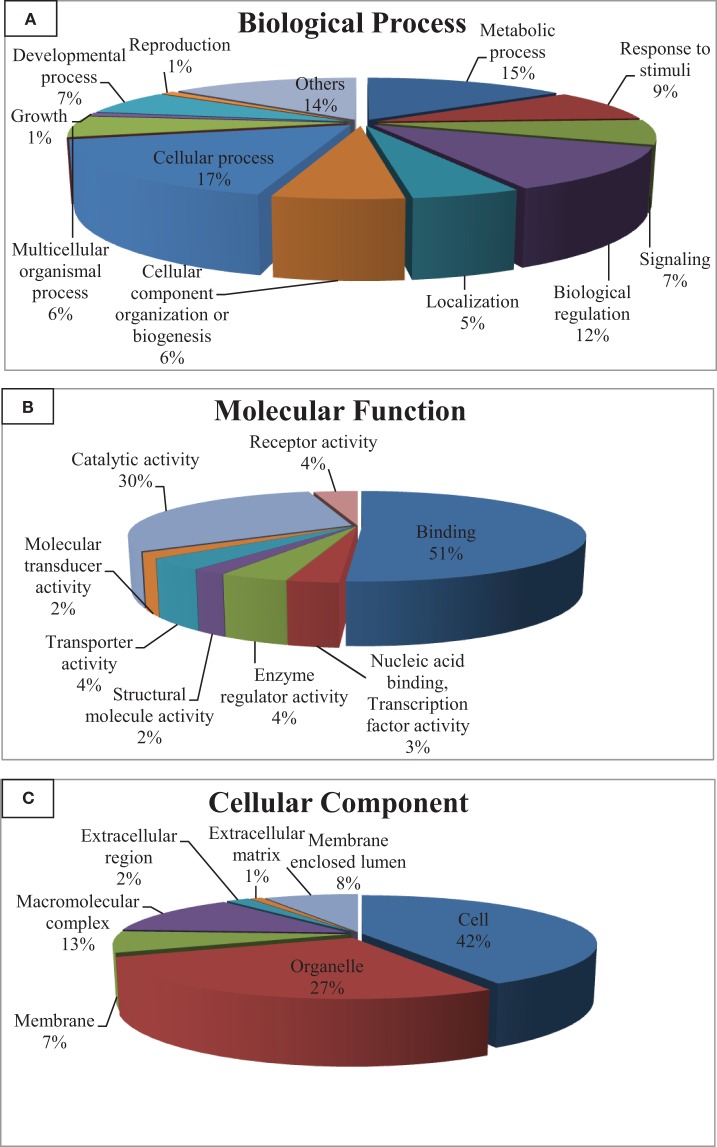
**Gene ontology (GO) assignments (2nd level GO terms) for the spleen transcriptomic sequences of rainbow trout. (A)** Refers to biological process, **(B)** refers to molecular function and **(C)** refers to cellular component.

**Figure 3:**
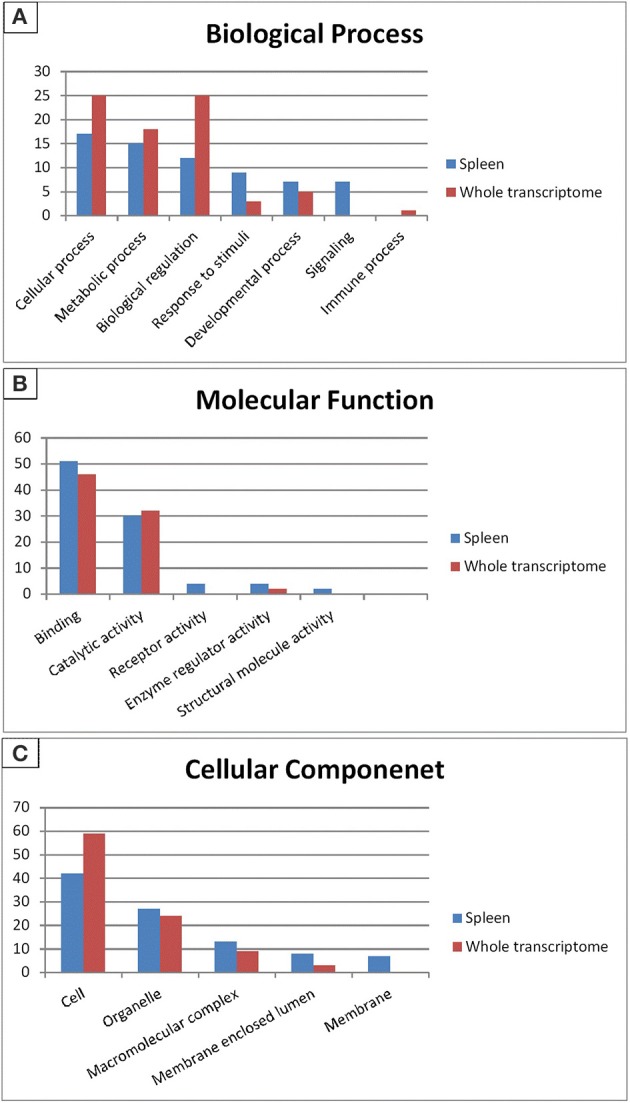
**Gene Ontology (2nd level GO terms) comparison of rainbow trout whole reference and spleen transcriptomes**. GO comparison shows a higher representation of signaling, response to stimuli, binding and receptor activity in spleen compared to whole reference transcriptome. **(A)** Refers to biological process, **(B)** refers to molecular function and **(C)** refers to cellular component.

Gene ontology (GO) categorizes gene products and standardizes their representation across species (Consortium, 2008). Contigs with lengths of 500 nucleotides or more (43,047) were annotated using the Blast2GO suite (Conesa et al., 2005; Götz et al., 2008); contigs were assigned to appropriate molecular function, biological process and cellular component GO terms (Ashburner et al., 2000). Figure 2 shows summary of the GO assignments at the second level in the three areas of gene ontology. GO term distribution was compared to a transcriptome reference that we previously assembled from 13 tissues (Salem et al., 2010) (Figure 3). In the biological process category, the most represented terms were related to cellular process (17%), followed by metabolic process (15%) and biological regulation (12%) (Figure 2A). These percentages are lower than the corresponding categories at the whole transcriptome reference; 25, 18, and 25%, respectively (Figure 3A). Conversely, some immune-relevant sub-categories of metabolic processes exist in higher percentages compared to counterparts at sub-categories at the whole transcriptome reference; response to stimuli (9%) and signaling (7%) compared to 3% response to stimuli and 1% immune system process, respectively (Figure 3A). These percentages suggest that we identified a larger number of immune-related genes in this study.

Within the molecular function category, 51, 30, and 4% of the spleen transcripts were assigned to binding, catalytic activity and receptor activity, respectively. The whole transcriptome reference showed 46 and 32% and unlisted percentage, respectively (Figure 3B). Pereiro and co-workers suggested several immune-related processes were represented in the binding and catalytic activity categories (Pereiro et al., 2012). In the cellular component categories, a significant percentage of clusters assigned to cell (42%), organelle (27%), macromolecular complex (13%), membrane enclosed lumen (8%) and membrane 7% (Figure 3C) compared to their corresponding categories at the whole transcriptome reference; 59, 24, 9, 3% and unlisted percentage, respectively (Figure 3C). Discrepancies in the GO distribution profiles may be attributed to differences in the nature of the cDNA libraries, the numbers of sequences used to retrieve GO terms, sequencing technology and the assembly approaches. Information about GO terms is supplied in additional Table S2.

KEGG pathway analysis was carried out to categorize and annotate the assembled contigs (Kanehisa and Goto, 2000; Kanehisa et al., 2012). Searching contigs against the KEGG database yielded 7,024 KEGG hits (16.32% of the total number of transcripts), with 4779 unique hits (11.10% of total number of transcripts). KEGG Orthology (KO) numbers were used to assign sequences to different metabolic pathways (Table 2). In total, 2236 (31.83% of the total number of KEGG hits) KEGG annotated sequences were assigned to metabolism that was further classified into carbohydrate metabolism (453 sequences, 20.26%), lipid metabolism (349 sequences, 15.61%) and amino acid metabolism (343 sequences, 15.34%). In addition, 1,394 (19.85%) annotated sequences were assigned to genetic information processing which includes folding, sorting and degradation (484 sequences, 34.72%), translation (459 sequences, 32.93%), replication and repair (255 sequences, 18.29%), and transcription (196 sequences, 14.06%). Further, 1830 sequences (26.05%) were classified as environmental information processing assigning 1508 sequences (82.40%) to signal transduction and 283 sequences (15.46%) to signaling molecules and interaction. The Cellular processes group contained 1421 (20.23%) KEGG-annotated sequences.

**Table 2:** **KEGG biochemical mappings for rainbow trout**.

**KEGG categories**	**No. of annotated sequences (%)**	**Number of KOs**
**Metabolism**	2236	1496
Carbohydrate metabolism	453 (20.26)	281
Lipid metabolism	349 (15.61)	220
Amino acid metabolism	343 (15.34)	239
Glycan biosynthesis and metabolism	273 (12.21)	187
Nucleotide metabolism	220 (9.84)	152
Energy metabolism	189 (8.45)	132
Metabolism of cofactors and vitamins	129 (5.77)	99
Metabolism of other amino acids	105 (4.70)	63
Xenobiotic biodegradation and metabolism	99 (4.43)	69
Biosynthesis of secondary metabolites	38 (1.70)	25
Metabolism of teroenoids and polyketides	38 (1.70)	29
**Genetic information processing**	1394	920
Folding, sorting and degradation	484 (34.72)	311
Translation	459 (32.93)	283
Replication and repair	255 (18.29)	196
Transcription	196 (14.06)	130
**Environmental information processing**	1830	1169
Signal transduction	1508 (82.40)	909
Signaling molecules and interaction	283 (15.46)	227
Membrane transport	39 (2.13)	33
**Cellular processes**	1421	905
Cell growth and death	485 (34.13)	306
Transport and catabolism	434 (30.54)	295
Cell communication	358 (25.19)	215
Cell motility	144 (10.13)	89
**Organismal systems**	2825	1777
Immune system	842 (29.81)	541
Endocrine system	619 (21.91)	378
Nervous system	526 (18.62)	334
Digestive system	268 (9.49)	166
Development	210 (7.43)	132
Excretory system	126 (4.46)	73
Circulatory system	103 (3.65)	69
Environmental adaptation	88 (3.12)	56
Sensory system	43 (1.52)	28
**Total**	9706	6267

Remarkably, the KEGG organismal systems category contained 2825 (40.22%) annotated sequences with the highest number of sequences assigned to immune system (842 sequences, 29.81%) followed by endocrine system (619 sequences, 21.91%), nervous system (526 sequences, 18.62%), digestive system (268 sequences, 9.49%) and development (210 sequences, 7.43%). Assignments of the organismal systems to the last four categories support the previously reported relationships between function of the spleen and other systems of the body. For example, a subset of genes with functions relevant to neurodevelopment was identified in the spleen transcriptome of the house finch (*Haemorhous mexicanus*) (Backström et al., 2013). Regarding the endocrine functions, it was thought that spleen secretes a hormone-like substance under the control of pituitary gland and adrenal cortex in case of emergencies (Ungar, 1945). Recently, the spleen endocrine function has been confirmed after in-depth studies of its function (Wu, 1998; Horiguchi et al., 2004; Tarantino et al., 2011). A cytokine known as Lymphotoxin was reported to keep the immunological balance of the gastrointestinal tract through regulation of the immune system of the digestive tract which is represented by immune cells, immunoglobulins and intestinal bacteria (Kruglov et al., 2013). In addition, hormones of the gastrointestinal tract activate the immune system in case of gut inflammation (Khan and Ghia, 2010).

### Taxonomic analysis

**Figure 4:**
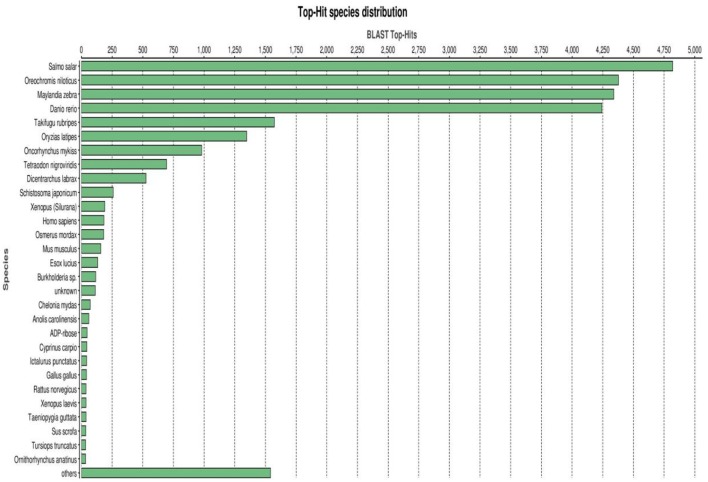
**BLASTX top-hit species distribution of gene annotations showing high homology to fish species with known genome sequences**.

A BLASTX top-hit species distribution of gene annotations showed highest homology to *Salmo salar* (4,833 BLAST hits; 18.35%), followed by *Oreochromis niloticus* (16.64%), *Maylandia zebra* (16.47%) and *Danio rerio* (16.12%) (Figure 4). Other fish species in the BLASTX top-hit were *Takifugu rubripes* (5.98%) and *Oryzias latipes* (5.17%). Rainbow trout itself (983 BLAST hits; 3.73%) fell in the seventh position of the top-hit species distribution. This may be explained by identification of a large number of new genes in this study and/or and existence of a limited number of rainbow trout proteins (6965 proteins) that currently available at NCBI database. The model fish species in the list, *D. rerio, T. rubripes* and *O. latipes*, have large number of proteins available in the NCBI database. All first nine species were fish, starting with *S. salar* from the family Salmonidae to which rainbow trout belongs. Therefore, these results support the high quality and high level of phylogenetic conservation of the assembled spleen transcriptome. Other species with known genome sequences appearing in the BLASTX top-hit species distribution were mammals including *Homo sapiens, Mus musculus*, and *Rattus norvegicus, Gallus* (chicken) and the amphibian *Xenopus laevis*.

### Immune transcriptome analysis

**Figure 5:**
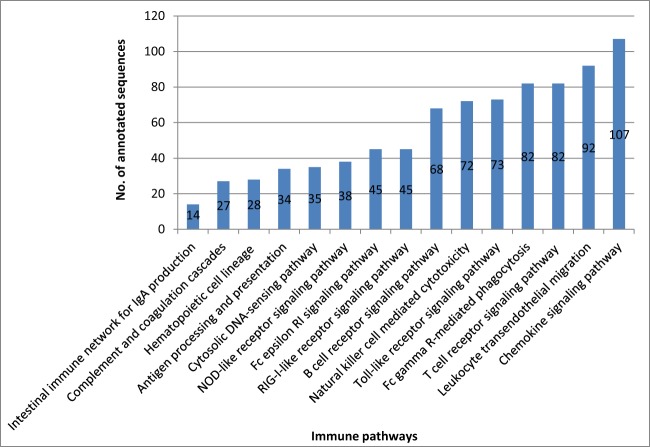
**Number of all annotated sequences obtained from the different KEGG immune pathway mapping**.

Our transcriptome analysis identified 842 immune-related transcripts in 15 KEGG immune pathways, (Table S3). Many of these transcripts are represented by complete cDNA sequences that were identified for the first time in rainbow trout. The immune-related transcripts were mapped to a newly assembled genome reference (Berthelot et al., 2014). The coordinate genome reference IDs and complete/incomplete ORF conditions are provided in Tables S4–S10. The immune-related transcripts were clustered according to their KEGG assigned pathways (Figure 5).

#### Toll-like receptor signaling pathway

Toll-like receptors (TLRs) activate the innate immune response through recognition of pathogen associated molecular patterns (PAMPs) including lipopolysaccharides or peptidoglycan in bacterial cell wall, β-1,3-glucan on fungal cell wall and dsRNA from viruses (Medzhitov and Janeway, 2000; Janeway and Medzhitov, 2002). This leads to the activation of nuclear factor- kB (NF-kB) that in turn induces proinflammatory cytokines (Barton and Medzhitov, 2003; Takeda and Akira, 2004).

More than 10 TLRs have been identified in teleost fish including zebrafish (Jault et al., 2004; Meijer et al., 2004), rainbow trout (Tsujita et al., 2004; Palti et al., 2010a), common carp (Kongchum et al., 2010), pufferfish (Oshiumi et al., 2003), channel catfish (Bilodeau and Waldbieser, 2005; Baoprasertkul et al., 2007a,b) and Atlantic salmon (Rebl et al., 2007). In our spleen transcriptomic data, there were 7 transcripts, as represented in Table S4, showing high similarity to TLR1, TLR2, TLR3, TLR5, TLR7/8, and TLR9. The coordinate genome reference IDs were identified and five transcripts matching TLR1, TLR3, TLR7, TLR8, and TLR9 had complete cDNA sequences. Several TLRs were previously identified in rainbow trout (Tsujita et al., 2004; Rodriguez et al., 2005; Ortega-Villaizan et al., 2009; Palti et al., 2010a,b). TLR4 was not reported in teleosts except zebrafish whereas TLR6 is totally missing in teleost fish (Takano et al., 2011). This study supports the notion of absence of both TLR4 and TLR6 in rainbow trout.

**Figure 6:**
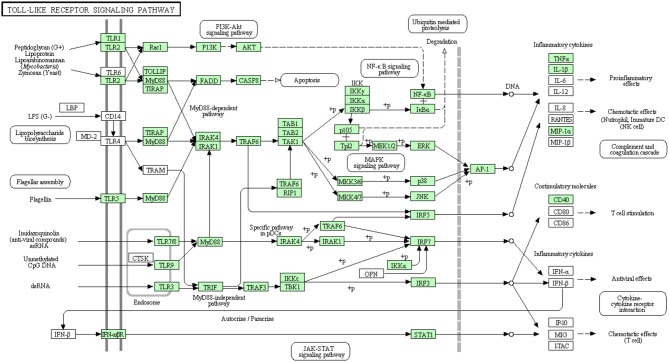
**Toll-like receptor signaling pathway showing the annotated and non-annotated proteins**. Proteins appearing in our transcriptome are represented in green color and absent proteins in white color.

There were three transcripts matching the NF-κ B complex. Two transcripts matched each of MKK4/7 and IFN-αβ R complexes. Each of MKK3/6 and MEK1/2 complexes has one transcript whereas MAP2K3 and MAP2K1 have no matches. Additionally, AP-1 which is composed of JUN and FOS (Zenz et al., 2008) had only one transcript matching JUN. The remaining 56 transcripts, out of the 73 total transcripts, showed high similarity to other members of the TLR signaling pathway of higher vertebrates (Figure 6). A total of 38 transcripts have complete cDNA sequences. To our knowledge, 26 different proteins were annotated for the first time in rainbow trout in the current study. Information about transcripts that showed homology to molecules involved in Toll-like receptor signaling pathway is included in additional Table S4.

#### B cell receptor signaling pathway

**Figure 7:**
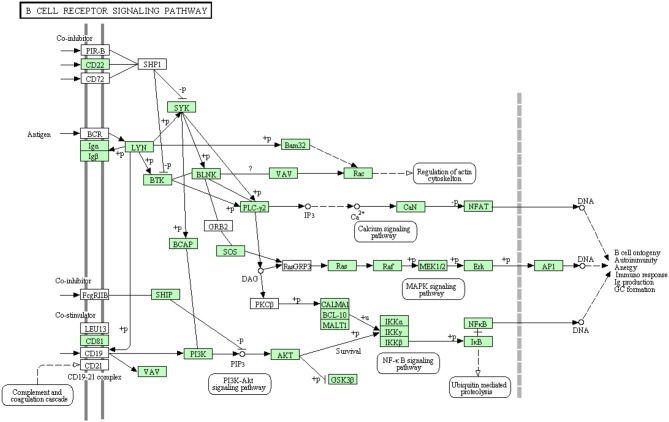
**B cell receptor signaling pathway showing the annotated and non-annotated proteins**. Proteins appearing in our transcriptome are represented in green color and absent proteins in white color.

B-lymphocytes are involved in antigen specific defense. They are activated through binding of antigen to B cell receptors. These cells produce specific antibodies to neutralize the foreign particles (Kurosaki et al., 2010). The binding of antigen to B-cell receptor activates B lymphocytes (Batista and Neuberger, 1998). Figure 7 shows all B cell signaling pathway annotated and non-annotated proteins in the current study. A total of 68 sequences were assigned to the B cell signaling pathway, 44 have been identified for the first time in this transcriptomic study. Information about transcripts that showed homology to molecules involved in B cell receptor signaling pathway is included in additional Table S5.

#### T cell receptor signaling pathway

**Figure 8:**
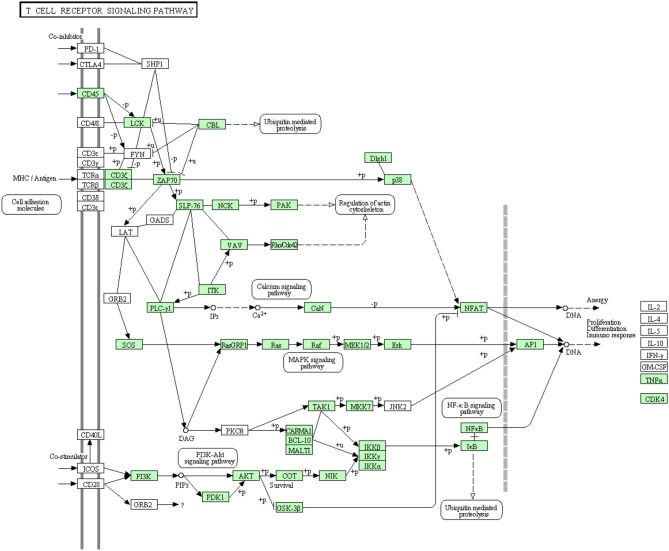
**T cell receptor signaling pathway showing the annotated and non-annotated proteins**. Proteins appearing in our transcriptome are represented in green color and absent proteins in white color.

Like B cells, T lymphocytes are involved in the antigen specific defense. Both T cell receptors (TCR) and costimulatory molecules such as CD28 are required for T cell activation. The cytotoxicity of Cytotoxic T lymphocytes in fish is obscure due to lack of suitable experimental systems even though few studies have depicted the lysis of virus-infected cells by NK-like cells in rainbow trout (Moody et al., 1985; Yoshinaga et al., 1994) and channel catfish (Hogan et al., 1996). Information about proteins that are involved in this cascade was very limited in rainbow trout. The annotated transcripts showed high similarity to many members of the T cell receptor signaling pathway of higher vertebrates as shown in Figure 8. In this study, many transcripts (56 out of 82) that are included in T cell receptor signaling pathway were identified for the first time. Information about transcripts that showed homology to molecules involved in T cell receptor signaling pathway is included in additional Table S6.

#### Chemokine Signaling Pathway

**Figure 9:**
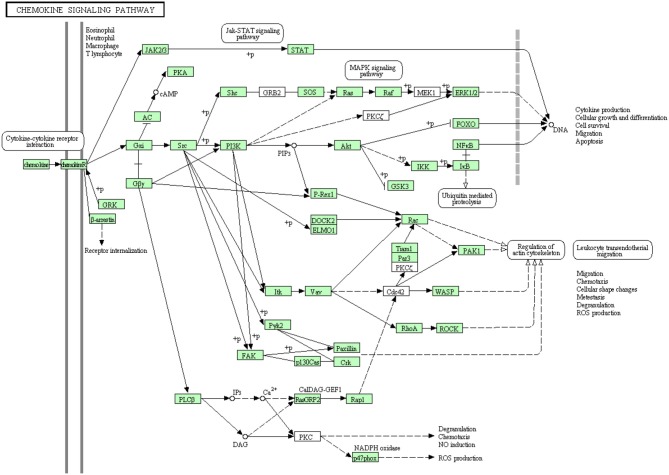
**Chemokine signaling pathway showing the annotated and non-annotated proteins**. Proteins appearing in our transcriptome are represented in green color and absent proteins in white color.

Chemokines have a major role in trafficking and activation of leukocytes toward the site of inflammation by the aid of C-terminal domain of their receptors (chemotaxis) (Pease and Williams, 2006). Few chemokine-related genes have been cloned in rainbow trout including CCR9 (Dixon et al., 2013), CXCR4, CCR7 (Daniels et al., 1999) and CXCL14 (Bobe et al., 2006). Many of chemokine receptors haven't been reported to date in rainbow trout (Dixon et al., 2013). In the present study, most of the proteins in the chemokine signaling pathway have been identified (Figure 9). Out of 107 annotated transcripts, 73 sequences matching 49 proteins have been identified for the first time in rainbow trout. Information about transcripts that showed homology to molecules involved in chemokine signaling pathway was included in additional Table S7.

#### Fc gamma R-mediated phagocytosis

**Figure 10:**
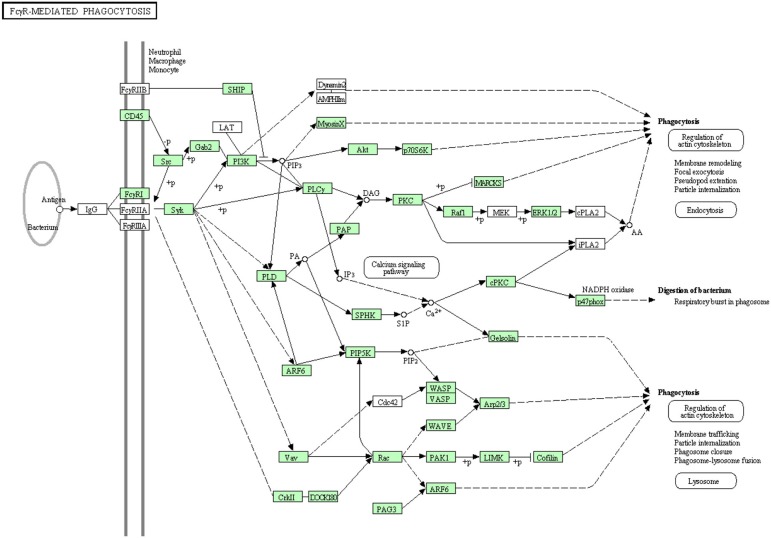
**Fc gamma R-mediated phagocytosis showing the annotated and non-annotated proteins**. Proteins appearing in our transcriptome are represented in green color and absent proteins in white color.

Clusters of IgG coat the foreign particles in a process termed opsonization. Leukocytes and tissue macrophages phagocytose the opsonized pathogens through Fc gamma receptors (Pacheco et al., 2013). Before the present study, some proteins were known to be involved in Fc gamma R-mediated phagocytosis as listed in Table S8. In the current transcriptome analysis, all annotated proteins belonging to the Fc gamma R-mediated phagocytosis are shown in Figure 10. There were 52 sequences out of 82 annotated sequences matching 30 proteins identified for the first time in rainbow trout. Information about transcripts that showed homology to molecules involved in the Fc gamma R-mediated phagocytosis signaling pathway is included in additional Table S8.

#### Leukocyte transendothelial migration

**Figure 11:**
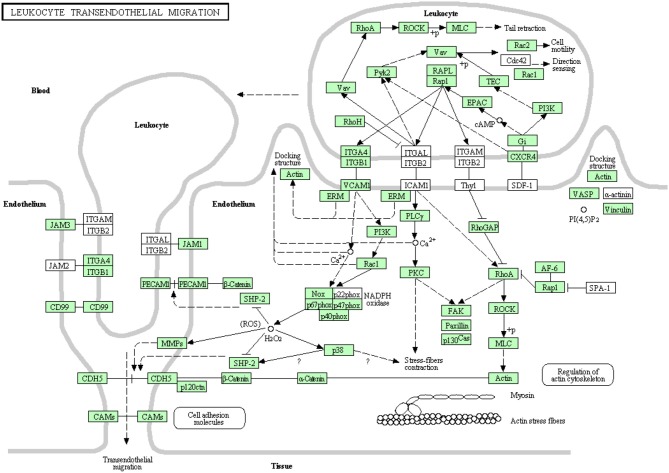
**Leukocyte transendothelial migration showing the annotated and non-annotated proteins**. Proteins appearing in our transcriptome are represented in green color and absent proteins in white color.

White blood cells migrate in an amoeboid fashion through the endothelium lining of the blood vessels to drive the immune response to the site of infection (Muller, 2011, 2013). Most of the proteins belonging to this pathway have been identified in this transcriptome analysis (Figure 11). There were 92 transcripts showing high similarity to members of the leukocyte transendothelial migration cascade of higher vertebrates. Prior to the current transcriptome sequencing, some proteins were previously annotated in rainbow trout (Table S9). To our knowledge, 36 proteins belonging to this pathway were reported for the first time in the current study. Information about transcripts that showed homology to molecules involved in leukocyte transendothelial migration is included in additional Table S9.

#### Natural killer (NK) cell mediated cytotoxicity

**Figure 12:**
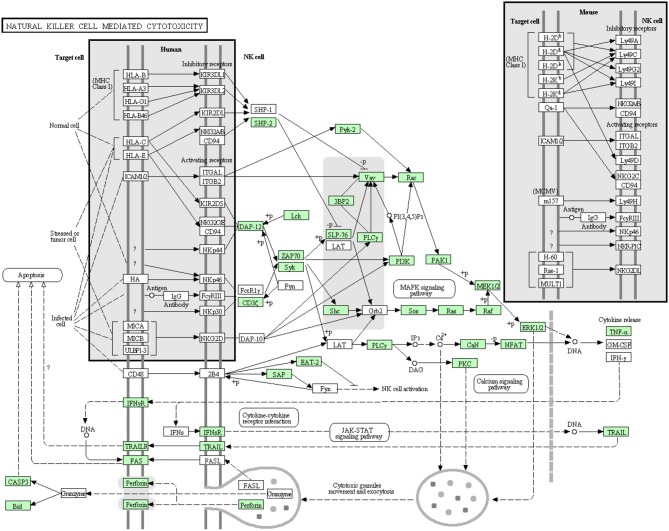
**Natural killer cell mediated cytotoxicity showing the annotated and non-annotated proteins**. Proteins appearing in our transcriptome are represented in green color and absent proteins in white color.

NK cells are lymphocytes working as a part of the innate immune system. Although they don't have classical antigen receptors like T and B lymphocytes, their receptors can discriminate between self and non-self-cells (Lanier, 2003). In the current transcriptome analysis, many but not all proteins that are involved in the NK cell mediated cytotoxicity pathway were annotated (Figure 12). Many proteins involved in this pathway were reported before the current study. In this cascade, 42 transcripts out of 72 identified sequences have been annotated for the first time in rainbow trout. The newly annotated transcripts matched 24 proteins. Information about transcripts that showed homology to molecules involved in NK cell mediated cytotoxicity signaling pathway is included in additional Table S10.

### Spleen-specific genes

Recently, a total of 51 spleen-specific have been identified in our lab (data will be published elsewhere). The assembled contigs were submitted to the NAGRP Aquaculture Genome Projects (http://www.animalgenome.org/repository/pub/MTSU2014.0811/). The coordinate IDs of the spleen-specific transcripts were determined using BLASTN (cut off *E*-value of 1.00E-10) against our spleen transcriptome (Table S11). As shown in Table S11, the level of gene expression was at least 20 fold higher in the spleen compared to 12 other tissues, with statistical false discovery rate (FDR) less than 5%. The spleen-specific genes were mapped to the newly assembled genome reference (Berthelot et al., 2014). The coordinate genome reference IDs and complete/incomplete ORF conditions are provided in additional Table S11.

The list of spleen-specific genes includes: (1) Immune proteins include 5 transcripts such as Fc receptor-like protein 3-like, thrombopoietin receptor precursor, P-selectin precursor, nuclear factor, interleukin 3 regulated (NFIL3), and lectin precursor. (2) Respiratory gas transport proteins include 4 most highly expressed transcripts assigned to hemoglobin and one transcript assigned to carbonic anhydrase II. A large representation of iron-binding proteins was reported in spleen of virus infected Turbot (Pereiro et al., 2012). (3) Coagulation cascade and adhesion proteins include 10 transcripts; five out of the ten transcripts were assigned to platelet glycoproteins whilst the other 5 transcripts were assigned to coagulation factor XIII A chain precursor, thrombopoietin receptor precursor, integrin alpha 2b, integrin beta-3-like and Von Willebrand factor. Thrombocytes appear in spleen during the first week postfertilization before appearing in blood because they participate in body defense (Tavares-Dias and Oliveira, 2009). (4) Development proteins involve 5 transcripts including ectodysplasin receptor, T-box transcription factor TBX6, homeobox proteins Nkx-2.6-like, T-cell leukemia homeobox protein 1 and Zinc finger protein Gfi1b. The participation of the house finch (*H. mexicanus*) spleen transcriptome in neurodevelopment through a subset of genes has been already reported (Backström et al., 2013). (5) Transporter proteins include one transcript for band 3 anion exchange protein. Both of the biosynthetic and membrane incorporation processes of Band 3 protein have been studied *in vivo* in erythroid spleen cells (Sabban et al., 1981). Among the other identified spleen-specific genes was 5-aminolevulinate synthase erythroid specific mitochondrial precursor which is necessary for heme biosynthesis. Other spleen-specific genes such as rhomboid-like protease 4, Methionine aminopeptidase 2, zinc finger protein 143-like, GATA binding factor 1, N-acetyltransferase 6-like, RING finger protein 151 and cytosolic purine 5′-nucleotidase were also expressed. In mouse and rat, 39 spleen-specific genes are found in tissue-specific database (Xiao et al., 2010). Moreover, 168 Refseq are preferentially expressed in human spleen based on ESTs (Liu et al., 2008). Further work is still needed to validate spleen-specific genes obtained from our high-throughput spleen transcriptome analysis.

**Figure 13:**
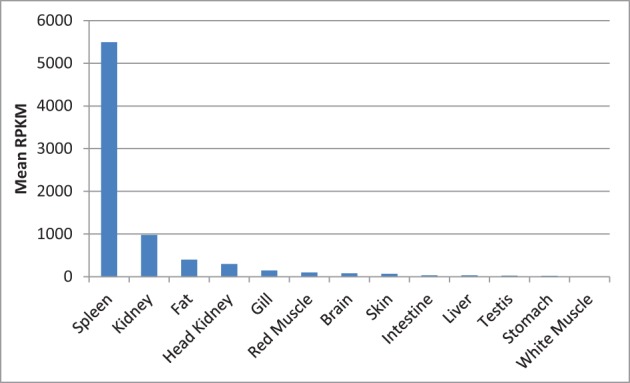
**Mean of RPKM of the spleen-specific genes in 13 tissues of rainbow trout**. The mean RPKM values of the spleen-specific genes were 5491, 973, and 400 in spleen, kidney and fat, respectively.

Next to spleen, the spleen-specific genes showed relatively higher expression in kidney and fat compared to the rest of the tissues. The mean RPKM values of the spleen-specific genes were 5491, 973, and 400 in spleen, kidney and fat, respectively (Figure 13). Kidney is one of the large lymphoid organs in teleosts containing macrophages and lymphoid cells (Zapata et al., 2006; Uribe et al., 2011). The relatively high level of expression of the spleen-specific genes in adipose tissue may be attributed to presence of many populations of immune cells in fat tissues (Ferrante, 2013).

Most of the 51 spleen-specific genes have functions not related to immunity. Interestingly, upon testing a few of these genes after infection with flavobacterium, three transcripts showed differential expression associated with infection, these are contig C23964_c2_seq1_P-selectin_precursor, contig C13_c2_seq1_Hemoglobin_subunit_beta-1 and contig C83628_c0_seq1_RING_finger_protein_151 (Detailed data will be published elsewhere). These data indicate that many of the spleen-specific genes may have immune functions. Further research is still needed to test if the other spleen-specific gene functions are related to immunity.

### ORF/ full-length cDNA prediction

Full-length cDNAs are a crucial tool for many genetic and genomic studies including alternative splicing and characterization of gene duplications or pseudogenes (Xin et al., 2008). To identify full-length cDNAs in the above mentioned seven immune pathways, contigs were analyzed by the TargetIdentifier server (Min et al., 2005). A total of 38, 29, 30, 49, 31, 37, and 26 full-length cDNAs were identified in the Toll-like receptor signaling pathway, B cell receptor signaling pathway, T cell receptor signaling pathway, chemokine signaling pathway, Fc gamma R-mediated phagocytosis, Leukocyte transendothelial migration and NK cell mediated cytotoxicity, respectively (Tables S4–S10). Out of the total number of full-length cDNAs, there were 25, 15, 18, 30, 24, 24, and 19 sequences with completely sequenced ORF identified in all studied immune pathways, respectively. Likewise, spleen-specific genes were analyzed by the online TargetIdentifier server (Min et al., 2005) to identify full-length cDNAs. A total of 37 full-length sequences including 24 with completely sequenced ORF were identified among the spleen-specific transcripts. Many of these transcripts were annotated for the first time in rainbow trout. Further work is needed to validate these full-length cDNAs and examine their genomic characteristics (UTR length, Kozak sequence, and conserved motifs) in detail.

## Methods

### Tissue sampling and RNA isolation

Homozygous doubled haploid rainbow trout fish from the Swanson clonal line were produced at Washington State University (WSU) by androgenesis (Scheerer et al., 1986, 1991; Young et al., 1996; Robison et al., 1999). The fish, approximately 300 g in weight, was reared in recirculating water systems at 12–16°C and had sexually matured as a male prior to sampling. Spleen tissues were collected and frozen in liquid nitrogen, then shipped on ice. All samples were preserved at −80°C until RNA isolation to reduce autocatalytic degradation. Total RNA was isolated from spleen tissues with TRIzol (Invitrogen, Carlsbad, CA) and purified according to the manufacturer's guidelines. Quantity of total RNA was assessed by measuring the absorbance at A260/A280 using a Nanodrop™ ND-1000 spectrophotometer (Thermo Scientific). RNA quality was checked by electrophoresis through a 1% (w/v) agarose gel. Moreover, RNA integrity was tested using the bioanalyzer 2100 (Aglient, CA).

### cDNA library preparation and illumina sequencing

RNA-Seq library preparation and sequencing were carried out by the University of Illinois at Urbana-Champaign (UIUC), 901 West Illinois street Urbana, IL 61801 USA. A RiboMinusTM Eukaryote Kit V2 (Invitrogen, Carlsbad, CA) was used to deplete rRNA from the total RNA. cDNA libraries were constructed using ~1 μg of rRNA depleted RNA following the protocol of the Illumina TruSeq RNA sample preparation Kit (Illumina). The resulting double-stranded manufactured cDNA was used in the preparation of the Illumina library. The standard end-repair step was carried out first, followed by the standard ligation reaction where the end-repaired DNA along with a single A base overhang were ligated to the adaptors using T4-DNA Ligase (TrueSeq RNA Sample Prep Kit v2, Illumina, San Diego, CA). The products of the ligation reaction were purified and exposed to size selection of the target length (400–450 bp) from the gel for carrying out the ligation-mediated PCR. Cluster generation and sequencing were carried out following the cluster generation and sequencing manual from Illumina (Cluster Station User Guide and Genome Analyzer Operations Guide). All sequenced raw data were first exported in FASTQ format and are currently being uploaded to the NCBI short read archive (SRA).

### CLC genomics *De novo* assembly

De novo assembly of the expressed short reads was carried out by CLC Genomics Workbench (version 6.0; CLC bio, Aarhus, Denmark; http://www.clcbio.com/products/clc-genomics-workbench/). The raw data were filtered by removing short, duplicated and low quality reads. CLC was run using the default settings for all parameters including a minimum contig length of 500 bp.

### Functional annotation and gene ontology analysis

Blast2Go version 2.6.5 (http://www.blast2go.com/b2ghome) was used for the functional annotation and analysis of the assembled contigs according to molecular function, biological process and cellular component ontologies. BLASTX search for sequence homology (*E*-value of 1.0E-3, maximum 20 hits) was carried out against NCBI's non-redundant protein database (NR). GO terms related to the established hits were extracted and modulated. The functional annotations were analyzed and statistical analysis of GO distributions was performed.

### Identification of immune-related proteins

Assembled consensus sequences were uploaded to the KEGG Automatic Annotation Server (KAAS) (Moriya et al., 2007) Ver. 1.67x (http://www.genome.jp/tools/kaas/). The functional annotation of genes was carried out by searching local BLAST against KEGG database.

The Bi-directional best hit (BBH) method was used to analyze and identify the immune molecules that were present and absent in the seven immune pathways containing the highest number of transcripts that showed high similarity to different members of each pathway. Transcripts were mapped to a newly assembled genome reference. The coordinate genome reference IDs of the immune-related transcripts were determined using BLASTX (cut off *E*-value of 1.00E-10) against the genome protein dataset (Berthelot et al., 2014).

### Identification of spleen-specific genes

Tissue-specific genes in spleen were identified using CLC genomics workbench Baggeley's test in which expression level of a gene in spleen was compared to its expression level in 12 tissues (brain, white muscle, red muscle, fat, gill, head kidney, kidney, intestine, skin, stomach, liver and testis). For distinction of tissue-specific genes, FDR value was set as 5% and fold change was at least 20 fold higher in spleen relative to the other 12 tissues. The spleen-specific genes were mapped to the newly assembled genome reference (Berthelot et al., 2014) to determine the coordinate genome reference IDs with a cutoff *E*-value of 1.00E-10.

### ORF/ full-length cDNA prediction

All contigs annotated in the interesting KEGG immune pathways, including Toll-like receptor signaling pathway, T-cell receptor signaling pathway, B-cell receptor signaling pathway, chemokine signaling pathway, Fc gamma R-mediated phagocytosis, Leukocyte transendothelial migration and NK cell mediated cytotoxicity, in addition to spleen-specific genes were analyzed using the online TargetIdentifier server (Min et al., 2005) to look for open reading frames and putative full-length cDNAs. A BLASTX output file including the BLASTX results for all cDNA sequences in the “FASTA” file with a cutoff *E*-value of 1.00E-3 was uploaded to the TargetIdentifier program to work properly. cDNA was considered as full-length if the sequence has a 5′ stop codon followed by a start codon or the sequence does not have a 5′ stop codon but there is an in-frame start codon present prior to the 10^th^ codon of the subject sequence. Based on the BLASTX results, TargetIdentifier predicts existence of an open reading frame (ORF) completely sequenced or not.

### Conflict of interest statement

The authors declare that the research was conducted in the absence of any commercial or financial relationships that could be construed as a potential conflict of interest.
